# Hippocampal Neurogenesis Levels Predict WATERMAZE Search Strategies in the Aging Brain

**DOI:** 10.1371/journal.pone.0075125

**Published:** 2013-09-23

**Authors:** Joana Gil-Mohapel, Patricia S. Brocardo, Will Choquette, Russ Gothard, Jessica M. Simpson, Brian R. Christie

**Affiliations:** 1 Division of Medical Sciences, Island Medical Program, University of Victoria, Victoria, British Columbia, Canada; 2 Department of Biology, University of Victoria, Victoria, British Columbia, Canada; 3 Department of Psychology, University of Victoria, Victoria, British Columbia, Canada; 4 Brain Research Centre and Program in Neuroscience, University of British Columbia, Vancouver, British Columbia, Canada; 5 Department of Cellular and Physiological Sciences, University of British Columbia, Vancouver, British Columbia, Canada; National Institute on Aging Intramural Research Program, United States of America

## Abstract

The hippocampus plays a crucial role in the formation of spatial memories, and it is thought that adult hippocampal neurogenesis may participate in this form of learning. To better elucidate the relationship between neurogenesis and spatial learning, we examined both across the entire life span of mice. We found that cell proliferation, neuronal differentiation, and neurogenesis significantly decrease with age, and that there is an abrupt reduction in these processes early on, between 1.5-3 months of age. After this, the neurogenic capacity continues to decline steadily. The initial abrupt decline in adult neurogenesis was paralleled by a significant reduction in Morris Water Maze performance, however overall learning and memory remained constant thereafter. Further analysis of the search strategies employed revealed that reductions in neurogenesis in the aging brain were strongly correlated with the adoption of spatially imprecise search strategies. Overall, performance measures of learning and memory in the Morris Water Maze were maintained at relatively constant levels in aging animals due to an increase in the use of spatially imprecise search strategies.

## Introduction

The ability to generate new neurons continues into adulthood in a few select regions of the mammalian brain, including the sub-ventricular zone (SVZ) and the sub-granular zone (SGZ) of the dentate gyrus (DG) of the hippocampus [1-6,7,8]. In the DG, newborn neurons must migrate a short distance from the SGZ to the granule cell layer (GCL) where they integrate into the existing circuitry [8,9]. The maturation of these neurons into functional units appears to occur over a period of four to five weeks following the initial mitotic event [9,10].

The hippocampus plays an integral role in the consolidation of declarative memory, as well as context-dependent and spatial learning processes [11,12] in both humans [13,14] and rodents [15-18]. New hippocampal neurons are believed to contribute to the functioning of the hippocampus and there is evidence that they are recruited into hippocampal neuronal circuits with spatial learning [19] and possess particular physiological properties that make them more susceptible to behavioral-dependent synaptic plasticity [20,21,22]. Thus, it is reasonable to speculate that these new neurons might be integral for hippocampal-dependent learning and memory [22,23]. In agreement with this hypothesis, numerous correlative studies have shown that hippocampal neurogenesis can be modulated by learning and behavioural experience [24-27], and that a loss in hippocampal neurogenic function can adversely affect memory formation [20,28-31].

The phases of adult neurogenesis are tightly regulated and can be influenced by physiological, pathological, and behavioral factors. For example, antidepressant drugs [32,33]; growth factors [34,35]; diet [36,37]; physical exercise [24,38,39]; environmental enrichment [40]; and learning [25] are known to up-regulate neurogenesis in the adult mammalian brain. Conversely, neurogenesis can be decreased by neurodegenerative processes [41,42]; inflammation [43]; stress [44]; and increased oxidative stress [45-47]. However, the most dramatic reductions are associated with the process of ageing [6,48-50].

In this study we used immunohistochemical techniques to evaluate all phases of the neurogenic process (cell proliferation, neuronal differentiation, cell survival, and overal neurogenesis) throughout the lifespan of C57Bl6/J mice (from 1 to 24 months of age). Additionally, we determined how age affects learning and memory abilities by testing 1.5-, 3-, 6-, 9-, and 12-month old mice in the MWM task. We observed that neurogenesis, learning, and memory, all peak early in odulthood, at around 1.5 months of age. Thereafter, while neurogenesis continues to decrease steadly with age, learning and memory abilities remain relatively constant throught the mice’s lifespan. However, when performing a detailed analysis of the search strategies used during the MWM training, we observed a strong correlation between the levels of overall neurogenesis and the types of strategies used, with younger mice with increased levels of neurogenesis preferentially adopting more spatially precise learning strategies and older mice with reduced levels of neurogenesis progressively adopting more spatially imprecise search strategies. This correlation suggests that levels of neurogenesis might predict the types of search strategies employed while learning a spatial task.

## Materials and Methods

### Animals

To evaluate endogenous hippocampal cell proliferation and differentiation we used proopiomelanocortin (POMC) – enhanced green fluorescent protein (EGFP) transgenic mice, which selectively express EGFP in newly born granule cells of the DG under the transcriptional control of POMC genomic sequences [51], and their wild-type littermate controls (background strain: C57Bl/6J). A colony of POMC-EGFP transgenic mice was established at the University of Victoria by cross-breeding POMC-EGFP heterozygous males with heterozygous females. The transgene is expressed in both heterozygous and homozygous animals and its expression was confirmed by polymerase chain reaction (PCR) using DNA obtained from tail snips [51]. Upon weaning, mice were group-housed by sex in groups of 5 mice/cage. POMC-EGFP-expressing male and female mice were sacrificed at 1, 1.5, 2, 2.5, 3, 4, 6, 12, 18, and 24 months of age. For each age group approximately equal numbers of males and females were used.

Additionally, we used C57Bl/6J male mice (Charles River, Quebec, Canada) to evaluate overall neurogenesis and learning and memory in non-transgenic animals. Only males were used for this set of experiments to avoid the potentially confounding effects on behavioural performance of hormonal alterations that are observed during the female estrous cycle and throughout the female life spam (i.e., from puberty to old age) [52]. Mice arrived at the University of Victoria’s Animal Care Unit single-housed and were allowed two weeks to acclimatize to the new facility before testing commenced. To avoid male dominance-related behaviors, these mice remained single-housed throughout the duration of the study. Animals were used for experiments either at 1.5, 3, 6, 9, or 12 months of age.

The criteria used to determine the initial time-point for the various temporal analyses performed with both C57Bl/6J naïve mice and POMC-EGFP transgenic mice (with C57Bl/6J background), was sexual maturation. Although C57Bl/6J mice continue to grow until about 3 months of age, they become sexually mature by 35 days [53]. Thus, for the purposes of this study and similarly to previous studies [48], early adulthood was defined between 1-1.5 months of age. All animals were maintained on a 12-hour light/dark cycle with constant ambient temperature and humidity. Food and water were available *ad libitum*. All animal procedures were conducted in accordance with the University of Victoria Animal Care Committee, and the Canadian Council for Animal Care policies.

### Assessment of endogenous hippocampal cell proliferation and neuronal differentiation

#### Tissue processing

POMC-EGFP-expressing mice were sacrificed at 1 [n = 9 (4 males + 5 females)], 1.5 [n = 10 (5 males + 5 females)], 2 [n = 8 (4 males + 4 females)], 2.5 [n = 8 (4 males + 4 females)], 3 [n = 10 (5 males + 5 females)], 4 [(n = 6 (3 males + 3 females)], 6 [(n = 6 (3 males + 3 females)], 12 [n = 7 (3 males + 4 females)], 18 [n = 11 (6 males + 5 females)], and 24 [n = 8 (4 males + 4 females)] months of age. Animals were deeply anesthetized with an intraperitioneal (i.p.) injection of urethane (250 mg/ml in water; 1.5 g/kg of body weight) and transcardially perfused with 0.9% sodium chloride (NaCl) followed by 4% paraformaldehyde (PFA). The brains were removed and left in 4% PFA overnight at 4 °C and then transferred to 30% sucrose. Following saturation in sucrose, serial coronal sections were obtained on a vibratome (Leica VT1000S, Nussloch, Germany) at 30 µm thickness. Sections were collected into a 1/6 section-sampling fraction and stored in a cryoprotectant solution [0.04 M Tris-buffered saline (TBS), 30% ethylene glycerol, 30% glycerol] at 4 °C. To assess the endogenous expression of EGFP by fluorescence microscopy, one series of brain sections was mounted onto 2% gelatin-coated microscope slides, coverslipped with polyvinyl alcohol (PVA) mounting medium with DABCO antifade (Sigma-Aldrich, St. Louis, MO, USA), and stored in the dark at 4°C.

#### Colorimetric immunohistochemistry

Two adjacent series of sections were processed for detection of the endogenous proliferative markers Ki-67, a nuclear protein that is expressed during all active phases of the cell cycle, but is absent from cells at rest [54,55] and proliferating cell nuclear antigen (PCNA), which is expressed during all active phases of the cell cycle and for a short period of time after cells become post-mitotic [55]. Briefly, after thorough rinsing, the sections were incubated in 10 mM sodium citrate buffer (in 0.1 M TBS, pH = 6.0) at 95°C for 5 minutes. This step was repeated twice in order to completely unmask the antigens. After quenching with 3% H_2_O _2_/10% methanol in 0.1 M TBS for 15 minutes and pre-incubating with 5% normal goat serum (NGS) for 1 hour, the sections were incubated for 48 hours at 4°C with a rabbit polyclonal anti-Ki-67 primary antibody (1:500; Vector Laboratories, Burlingame, CA, USA) or a rabbit polyclonal antibody against PCNA (1:100; Santa Cruz Biotechnology, Santa Cruz, CA, USA). After thorough rinsing the sections were then incubated for 2 hours with a biotin-conjugated goat anti-rabbit IgG secondary antibody (1:200; Vector Laboratories). The bound antibodies were visualized using an avidin-biotin-peroxidase complex system (Vectastain ABC Elite Kit, Vector Laboratories) with 3,3’-diaminobenzidine (DAB; Vector Laboratories) as a chromogen. The sections were mounted onto 2% gelatin-coated microscope slides, dehydrated in a series of ethanol solutions of increasing concentrations followed by a 5-minute incubation with a xylene substitute (CitriSolv, Fisher Scientific, Fair Lawn, NJ, USA), and coverslipped with Permount mounting medium (Fisher Scientific).

One additional series of sections was processed for detection of the neurogenic differentiation protein (NeuroD), a basic helix-loop-helix transcription factor involved in neuronal differentiation [56,57]. Briefly, after quenching and pre-incubation with normal horse serum (NHS), the sections were incubated for 48 hours at 4°C with a goat anti-NeuroD primary antibody (1:200; Santa Cruz Biotechnology). The sections were then incubated for 2 hours with a biotin-conjugated horse anti-goat IgG secondary antibody (1:200; Vector Laboratories), and the bound antibodies were visualized as described above.

### Assessment of overall hippocampal neurogenesis

#### BrdU administration and tissue processing

**Figure 1:**
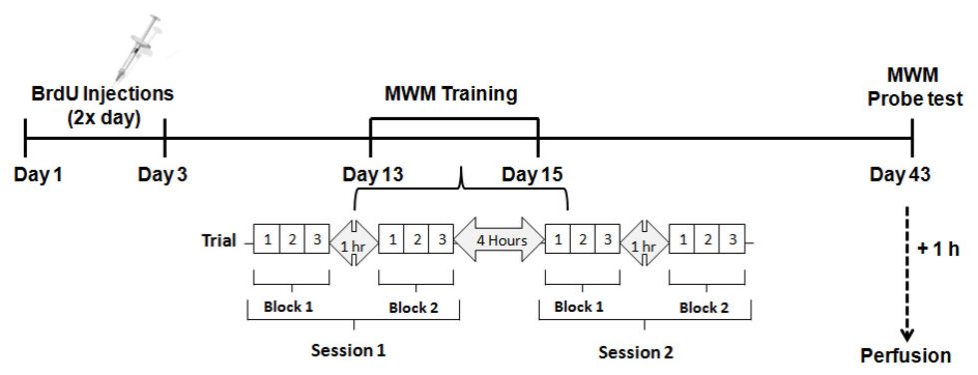
Experimental time-line for the BrdU and Morris water maze experiments. C57BL/6J male mice with 1.5, 3, 6, 9, and 12 months of age received BrdU injections (200 mg/kg i.p.) every 12 hours for 3 consecutive days. On day 12, animals were pre-trained in the MWM (three trials of 60 seconds each) with the platform protruding from the water surface. Between days 13 and 15 mice were trained to locate the hidden platform in the MWM. Each day animals received two training sessions separated by a 4-hour interval. Each session was composed by two blocks (of three 60-second trials each) separated by one hour. Mice were left undisturbed between days 16 and 42 and on day 43 they received a probe test in the MWM by placing them in the water without the platform for 60 seconds and assessing the amount of time they spent in the target quadrant (where the platform used to be located). Mice were sacrificed one hour later by transcardial perfusion and their brains were processed for immunohistochemistry.

We used 5-bromo-2’-deoxyuridine (BrdU) to evaluate overall neurogenesis. BrdU is a thymidine analogue that is incorporated into the DNA double-helix during the S-phase of the cell cycle, being a commonly used exogenously administered marker for cell proliferation and neurogenesis [58]. C57Bl/6J male mice at 1.5 (n = 8), 3 (n = 8), 6 (n = 7), 9 (n = 8), or 12 (n = 9) months of age received i.p. injections of BrdU (200 mg/kg, in 0.1 M TBS, pH = 7.2; Sigma-Aldrich) every 12 hours for 3 consecutive days. The same mice were then submitted to behavioral testing on the Morris water maze (MWM; see Section 2.5) and were sacrificed 42 days later (Figure 1) by transcardial perfusion with 0.9% NaCl followed by 4% PFA exactly as described above (Section 2.2). The brains were removed, left in 4% PFA overnight at 4 °C, and transferred to 30% sucrose. Following sucrose saturation, serial coronal sections were obtained exactly as described above (Section 2.2).

#### Fluorescence immunohistochemistry

Neuronal differentiation and overall neurogenesis were assessed by BrdU / neuronal nuclei (NeuN) / glial fibrillary acid protein (GFAP) triple-labeling. Briefly, after DNA denaturation in 2N HCl at 65°C for 30 minutes and pre-incubation with 5% NHS and 5% normal donkey serum (NDS) in 0.1 M TBS with 0.25% Triton X-100 (blocking solution), the sections were incubated for 48 hours at 4°C in rat anti-BrdU (1:100; Harlan Sera-Lab, Belton, UK), mouse anti-NeuN (1:100; Chemicon, Billerica, MA, USA), and rabbit anti-GFAP (1:800; Dako, Glostrup, Denmark) primary antibodies in 5% blocking solution (5% NHS and 5% NDS in 0.1 M TBS with 0.25% Triton X-100). The sections were then incubated with the secondary antibodies Cy3-conjugated donkey anti-rat IgG (1:200; Jackson ImmunoResearch, West Grove, PA, USA), Cy5-conjugated donkey anti-rabbit IgG (1:200; Jackson ImmunoResearch), and biotinylated horse anti-mouse IgG (rat absorbed) (1:200; Vector Laboratories) in 2% blocking solution (2% NHS and 2% NDS in 0.1 M TBS with 0.25% Triton X-100) for 2 hours, followed by incubation with Alexa-488-conjugated streptavidin (1:200; Molecular Probes, Leiden, The Netherlands) for 2 hours. The sections were mounted onto 2% gelatin-coated microscope slides, coverslipped with PVA mounting medium with DABCO antifade (Sigma-Aldrich), and stored in the dark at 4°C.

### Morphological quantification

#### Conventional and fluorescence microscopy

All morphological analyses were performed on coded slides, with the experimenter blinded to the identity of the samples, using an Olympus microscope (Olympus BX51, Center Valley, PA, USA) with 10x, 40x and 100x objectives. Image Pro-Plus software (version 5.0 for WindowsTM, Media Cybermetic Inc., Silver Spring, MD, USA) and a Cool Snap HQ camera (Photometrics, Tucson, AZ, USA) were used for image capture. Conventional microscopy was used for the analysis of Ki-67-, PCNA-, and NeuroD-labeled sections, whereas fluorescence microscopy was used for the analysis of BrdU- and EGFP-labeled section A modified stereological approach was used to estimate the total numbers of BrdU-, Ki-67-, PCNA-, NeuroD-, and EGFP-positive cells present along the entire SGZ of the DG of the hippocampus as previously described by us [59-61] and others [62-64]. All sections along the entire dorsal/ventral axis of the hippocampus that contained the DG sub-region (i.e., from 1.34 mm posterior to Bregma to 3.52 mm posterior to Bregma [65]) were used for the analysis, resulting in 9-10 DG sections per animal. All positive cells present along the entire SGZ of each DG section and located within two to three nuclear diameters below the GCL were counted. The results were expressed as the total number of labeled cells in the DG sub-region of the hippocampus by multiplying the average number of labeled cells/DG section by the total number of 30 µm thick-sections containing the DG (estimated to be 73 sections in the mouse brain). Images were processed with Adobe Photoshop 4.0 (Adobe Systems, Mountain View, CA, USA). Only contrast enhancements and color level adjustments were made; otherwise images were not digitally manipulated.

#### Confocal microscopy

Co-localization between BrdU and NeuN or BrdU and GFAP was assessed using a confocal laser-scanning microscope (Olympus BX61WI) connected to a PC running the Olympus FluoView FV10-ASW 1.7c Software, at 1 µm optical thickness. A maximum of 50 BrdU-labeled cells per mouse was randomly selected for analysis of co-localization with NeuN or GFAP. Previous studies have demonstrated that the migration of BrdU-labeled cells ends early after cell division and that by 4 weeks post BrdU incorporation, the majority of BrdU-labeled cells is located within the SGZ and the inner third of the GCL, indicating that after the first few weeks the surviving cells do not substantially change their position within the GCL [66]. Thus, all selected cells were located within the inner third of the GCL and the SGZ (i.e., within two to three nuclear diameters below the GCL). All sections containing the DG region of the hippocampus (9-10 DG sections/mouse; from 1.34 mm posterior to Bregma to 3.52 mm posterior to Bregma [65]; were used for the analysis with 2-5 BrdU-labeled cells per DG-containing section being randomly selected for imaging, thus ensuring a uniform analysis through the dorsal-ventral axis of this hippocampal sub-region. Co-localization between BrdU and NeuN was defined by nuclear co-localization of the two markers over the extent of the nucleus in consecutive 1 µm z-stacks, if profiles of green (NeuN) and red (BrdU) fluorescence coincided, and when co-localization was confirmed in *x-y, x-z* and *y-z* cross-sections produced by orthogonal reconstructions from z-series. GFAP is a filamentary protein expressed throughout the soma and filaments of astrocytes [67]. As such, BrdU and GFAP were considered to be co-localized when red nuclear fluorescence (BrdU) coincided with blue soma fluorescence (GFAP) over consecutive 1µm z-stacks, and when co-localization was confirmed in *x-y*, *x-z*, and *y-z* cross-sections produced by orthogonal reconstructions from z-series.

### Behavioral analysis: Morris water maze test

The MWM is a commonly used behavioral test to evaluate hippocampal-dependent spatial learning and memory in rodents [68-71]. In this study we used a modified version of the MWM protocol that was described by Kee and collaborators [19,72]. The MWM apparatus consists of a standard white circular swimming pool (100 cm diameter) filled with water maintained at 22°C. A transparent Plexiglas platform was positioned in the pool submerged 0.5 cm below the water surface. C57BL/6J male mice at 1.5 (n = 8), 3 (n = 8), 6 (n = 7), 9 (n = 8), or 12 (n = 9) months of age were used for these experiments (same male mice used for assessing overall hippocampal neurogenesis, as described in Section 2.3).

#### Pre-training

Nine days after receiving the last BrdU injection (see Section 2.3; Figure 1), each individual mouse received three training trials. For all trials, the mouse was placed in the pool facing the wall in a different starting point (NW, NE, SW, or SE) and was then allowed to swim for a maximum of 60 seconds or until it reached the platform. If the mouse failed to find the platform during the trial, it was manually guided to the platform by the investigator and placed on top of it for 15 seconds.

#### Assessment of learning

Testing commenced the day after pre-training and was done over a period of 3 consecutive days (Figure 1). Each day, mice received two testing sessions separated by approximately 4 hours. Each session consisted of 2 blocks (of 3 trials each) separated by 1 hour. For each trial, the animal was placed in the platform for a period of 15 seconds, then picked-up from the platform and placed in the pool facing the wall in a different starting point (NW, NE, SW, or SE). For each trial, the mouse was allowed to find the platform for a period of 60 seconds. When the mouse reached the platform, it was allowed to stay there for 15 seconds before being placed in its home cage. If the mouse failed to find the platform during the trial, it was manually guided to the platform by the investigator and placed on top of it for 15 seconds. The maximum duration of each trial and the interval between consecutive trials was 60 seconds. The platform remained in a fixed location throughout testing. The location of the platform remained constant for all mice.

#### Assessment of memory (probe test)

Twenty-eight days after testing, animals were submitted to a single probe trial to assess their spatial memory (i.e., their ability to remember where the platform was located during the training period). Each mouse was individually placed on the platform (located in the same position used during the training period) for 15 seconds, then picked up from the platform, the platform was removed from the pool, and the mouse was placed back in the pool in a unique start position (180° opposite the platform location). Finally the mouse was removed from the pool once 60 seconds had elapsed [72]. One hour after the start of the probe test mice were sacrificed as described in Section 2.3 (Figure 1).

#### Analysis of behavioral data

Behavioral data were acquired and analyzed using the AnyMaze automated video-tracking system (Stoelting Co., Wood Dale, IL, USA). The parameters used to assess learning included the latency to reach the platform (i.e., escape latency), and the search strategies employed in each trial. The predominant search strategy used in each trial was objectively classified with a criterion-based algorithm using Matlab (MathWorks, Natick, MA, USA) by a researcher blind to the mouse’s age. Trials were classified as follows (modified from [64,73,74]): (1) *direct swim*, characterized by a maintained heading towards the platform (<15 cm path length or heading <20° away from the platform at each 5 cm point starting at 15 cm path length); (2) *focal search*, characterized by a highly localized search near the platform (≥50% trial in 15 cm radius target zone, centered on the platform location); (3) *directed search*, characterized by a preference for a corridor towards the platform or platform quadrant (>80% trial within a 50 cm-wide corridor from start point to platform); (4) *chaining*, characterized by searching near the correct radial distance of the platform to the wall (>75% trial 20–50 cm from pool center, <15% within 10 cm of wall, and <10% within 20 cm of pool center); (5) *scanning*, characterized by a preference for the central pool area in which distal cue visibility is maximal (>50% trial within 35 cm of pool centre); (6) *thigmotaxis*, characterized by maintaining close proximity to the wall (>70% trial within 10 cm of wall); (7) *perseverance*, characterized by an erroneous preference for a non-target area (>60% trial in one or >75% in two adjacent non-target quadrant(s) and 750 cm path length); and (8) *random search*, characterized by no other classified strategy (remaining unclassified trials). Performance during the probe test was assessed by the amount of time mice searched the target zone (i.e., the quadrant where the platform used to be located), the number of times the animal crossed the platform zone (i.e., number of crossings). In order to control for any potential age-related deficits in locomotor activity, path length and speed were recorded for all trials.

### Statistical analysis

Statistical analyses were performed using Excel (Microsoft Office 2007, Mississauga, ON, Canada) and Statistica 7.1 analytical software (StatSoft Inc., Tulsa, OK, USA).

#### Immunohistochemical analyzes

To assess overall hippocampal neurogenesis in BrdU-treated male C57Bl/6J mice, data were analyzed with a one-factor analysis of variance (ANOVA), with age being the between-subjects factor for each data set. To assess endogenous hippocampal cell proliferation (i.e., number of Ki-67- and PCNA-positive cells) and differentiation (i.e., number of NeuroD- and EGFP-positive cells) in POMC-EGFP male and female mice, a two-factor ANOVA with age and sex as between-subjects factors revealed no significant main effect of sex [Ki-67: F(1, 44) = 0.34, *p* = 0.54; PCNA: F(1, 45) = 0.31, *p* = 0.86; NeuroD: F(1, 45) = 0.02, *p* = 0.89; EGFP: F(1, 43) = 1.63, *p* = 0.21], a finding that is in agreement with previous studies (Ben Abdallah et al., 2010). Thus, data from both males and females were combined and analyzed with one-factor ANOVAs with age as the between-subjects factor. Post-hoc analyses were conducted using the Tukey’s test. Changes in the numbers of Ki-67-, PCNA-, BrdU-, NeuroD-, and EGFP-positive cells with age were best fitted to an exponential model. Correlation coefficients (*r* ^*2*^) for the relationship between age and each one of the markers used were calculated. For the sake of clarity, only significant differences between subsequent age groups or, if an age group did not differ from the subsequent one, for the next group along the timeline that did differ, are highlighted in the figures.

#### Behavioral analyzes

The latency to find the hidden platform, the path length, and the speed were compared: (1) across the various age groups using factorial ANOVAs with age as the between-subjects factor; and (2) across the three days of training for each time-point using repeated measures ANOVAs. The difference between the average time required to locate the hidden platform on day 1 versus day 3 of training, and the number of crossings over the platform zone were compared across age groups using one-factor ANOVAs with age as the between-subjects factor. All post-hoc analyzes were conducted using the Tukey’s post-hoc test. Swim strategy frequencies were compared across trials within each age group using Friedman’s ANOVA and Kendall Coefficient of Concordance, whereas comparisons among age groups were assessed using Spearman Rank Order Correlations. The time each age group spent in the target quadrant during the MWM probe test was compared with the “chance” value of 15 seconds (i.e., the amount of time any animal would be expected to spend in the target quadrant if no spatial memory had been formed) with one-sample Student’s t-tests. The distance traveled during the first trial of the first day of training was compared with the distance traveled during the probe test using unpaired two-tailed Student’s t tests. Data are presented as means ± standard error of the mean (SEM). A *p* value of ≤ 0.05 was considered to be statistically significant. Correlation coefficients (*r* ^*2*^) for the relationship between the levels of overall neurogenesis and performance during the MWM test (use of spatially precise search strategies and performance during the prove test) were also calculated. Furthermore, the relationship among age, levels of overall neurogenesis, and the use of spatially precise search strategies or the performance during the probe test for each individual animal were analyzed by estimating the respective Pearson Correlation Coefficients (*r*).

## Results

### Hippocampal cell proliferation is drastically decreased by age

In order to further delineate the pattern of age-associated decrease in hippocampal proliferative capacity, we assayed DG cell proliferation in POMC-EGFP-expressing mice of the following ages: 1, 1.5, 2, 2.5, 3, 4, 6, 12, 18, and 24 months. In all animals, immunohistochemistry was performed for the endogenous proliferation markers Ki-67 and PCNA [55].

**Figure 2:**
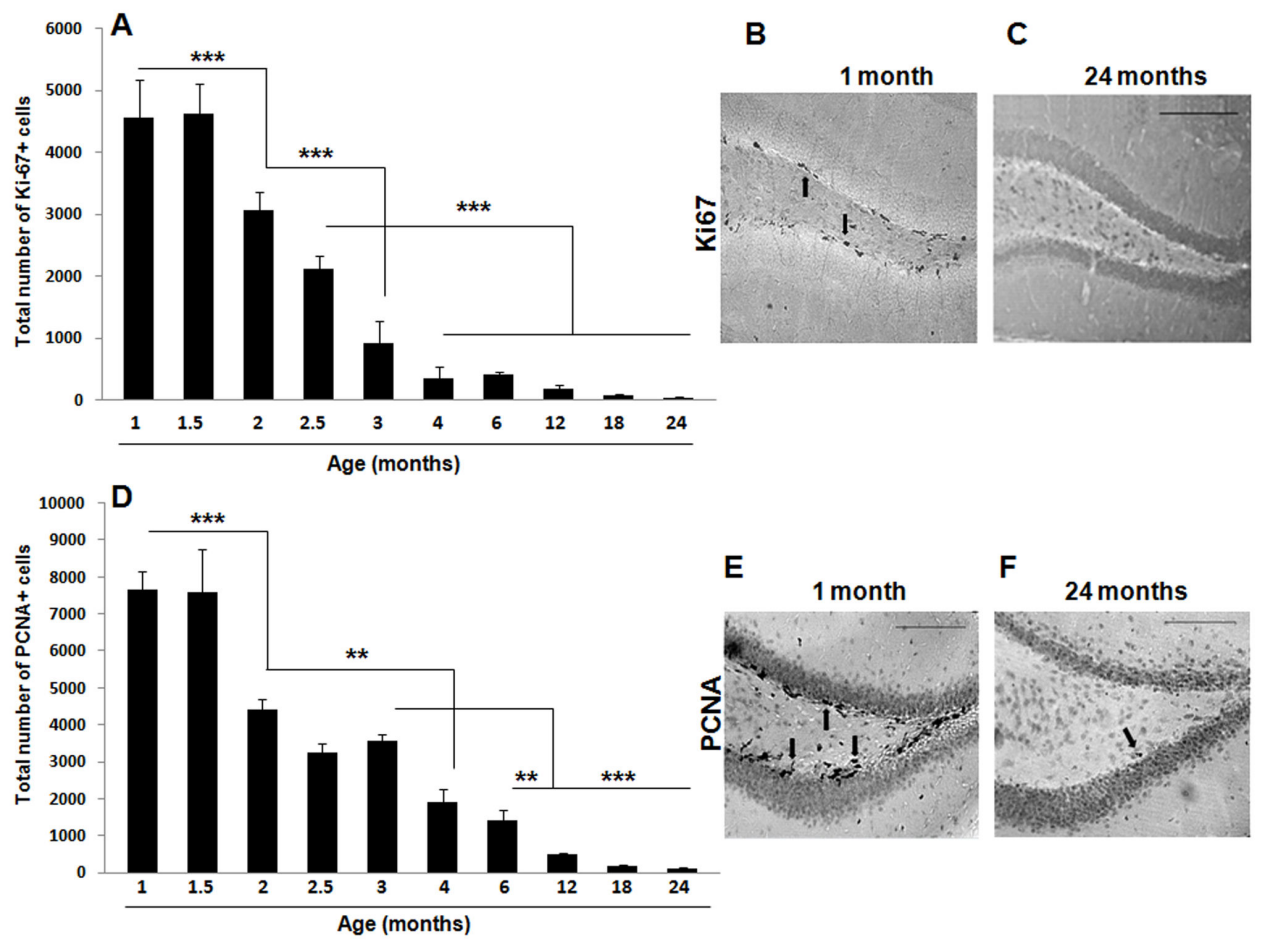
Cell proliferation is significantly decreased by age in the mouse hippocampal DG. POMC-EGFP transgenic mice were sacrificed at 1, 1.5, 2, 2.5, 3, 4, 6, 12, 18, 24 months of age by transcardial perfusion and their brains were processed for immunohistochemistry for the endogenous cell proliferation markers Ki-67 (A-C) and PCNA (D-F). Age induced a significant decline in the expression of both Ki-67 (A-C) and PCNA (D-F). An abrupt reduction in cell proliferation was detected early on (between 1.5 and 3 months of age) with both markers. Data are presented as means ± SEM. (A) *** *p* < 0.001 between 1 or 1.5 and 2 months, between 2 and 3 months, and between 2.5 and 4, 6, 12, 18 or 24 months. (D) ** *p* < 0.01 between 2 and 4 months, and between 3 and 6 months, *** *p* < 0.001 between 1 or 1.5 and 2 months, and between 3 and 12, 18, or 24 months. See text for additional statistical details. Representative sections of the DG processed for Ki-67 (B, C) and PCNA (E, F) immunohistochemistry in 1- (B, E) and 24- (C, F) month old POMC-EGFP mice. Black arrows indicate either Ki-67- (B) or PCNA- (E, F) immunopositive cells in the DG of 1- (B, E) and 24- (F) month old mice. Scale bars = 100 µm.

As expected, we observed a progressive decrease in the expression of Ki-67 in the DG with age [one-factor ANOVA, F(9, 68) = 55.43, *p* < 0.001] that was best fitted to a negative exponential curve [*Y* = 2733x e^(-0.199X)^, with a correlation coefficient *r* ^2^ = 0.85] (Figure 2 A-C). An abrupt four-fold decrease in the number of Ki-67 positive cells was detected very early on, between 1 (4550 ± 468 Ki-67-positive cells) and 3 (915 ± 279 Ki-67-positive cells) month-old animals (*p* < 0.001). The analysis of further time-points during early adulthood revealed a significant reduction in hippocampal cell proliferation between 1.5 and 2 months of age (*p* < 0.001) followed by a subsequent reduction that occurred between 2 and 3 (*p* < 0.001) and 2.5 and 4 (*p* < 0.001) months of age.

This progressive decrease in hippocampal cell proliferation was confirmed using a second proliferation marker, PCNA [one-factor ANOVA, F(9, 68) = 54.95, *p* < 0.001] (Figure 2 D-F). Again, the reduction in the number of PCNA-positive cells was best fit to a negative exponential curve [*Y* = 5976x e^(-0.181X)^, with a correlation coefficient *r* ^2^ = 0.94] and a drastic reduction in cell proliferation was detected between 1 (7656 ± 380 PCNA-positive cells) and 3 (3574 ± 126 PCNA-positive cells) months of age (*p* < 0.001). When additional time-points were analyzed, a significant reduction in the number of PCNA-positive cells was again detected between 1.5 and 2 months of age (*p* < 0.001). A further significant decrease in PCNA expression was detected between 2 and 4 (*p* < 0.01) and 3 and 6 (*p* < 0.01) months of age.

Taken together, these results indicate that a dramatic reduction in the endogenous proliferative capacity occurs very early on during adult life in the mouse DG (i.e., between 1 and 3 months of age). Nevertheless, although cell proliferation continues to decrease after that, some proliferating cells can still be detected even at 2 years of age (119 ± 16 Ki-67-positive cells and 35 ± 10 PCNA-positive cells).

### Age progressively decreases hippocampal neuronal differentiation

**Figure 3:**
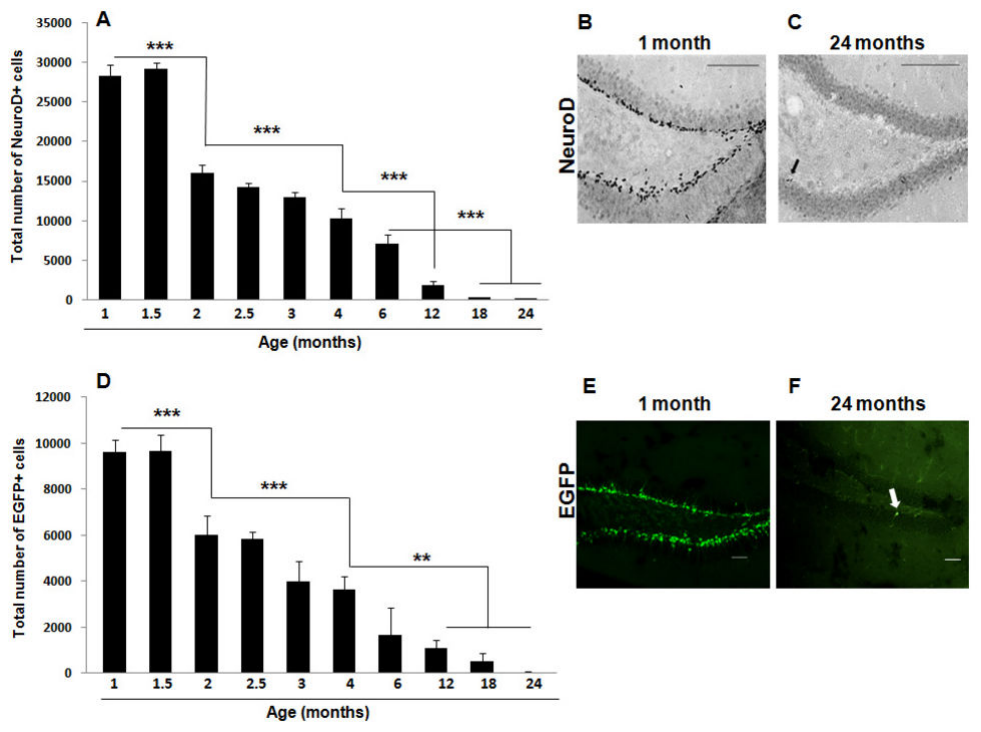
Neuronal differentiation is significantly decreased by age in the mouse hippocampal DG. POMC-EGFP-expressing mice were sacrificed at 1, 1.5, 2, 2.5, 3, 4, 6, 12, 18, 24 months of age by transcardial perfusion and their brains were processed for immunohistochemistry for the immature neuronal marker NeuroD (A-C) and visualized for the expression of EGFP (D-F). Age induced a significant decline in the expression of both NeuroD (A-C) and EGFP (D-F). An abrupt reduction in neuronal differentiation was detected early on (between 1.5 and 3 months of age) with both markers. Data are presented as means ± SEM. (A) *** *p* < 0.001 between 1 or 1.5 and 2 months, between 2 and 4 months, between 4 and 12 months, and between 6 and 18 or 24 months. (D) ** *p* < 0.01 between 4 and 12, 18, or 24 months, *** *p* < 0.001 between 1 or 1.5 and 2 months, and between 2 and 4 months. See text for additional statistical details. Representative sections of the DG processed for NeuroD immunohistochemistry (B, C) and analyzed for EGFP expression (E, F) in 1- (B, E) and 24- (C, F) month old POMC-EGFP mice. Arrows indicate a NeuroD-immunopositive cell (C) and an EGFP-expressing cell (F) in the DG of a 24-month old mouse. Scale bars (B, C) = 100 µm; scale bars (E, F) = 50 µm.

POMC-EGFP transgenic mice selectively express EGFP in newly born DG granule cells [51], and thus EGFP can be used as an endogenous neuronal differentiation marker in these mice. In agreement with the proliferation results (Figure 2), there was a progressive decrease in the expression of EGFP in the DG with age [F(9, 60) = 58.37, *p* < 0.001] that was best fitted to a negative exponential curve [*Y* = 9467.6x e^(-0.2X)^, with a correlation coefficient *r* ^2^ = 0.94] (Figure 3 D-F). There was also an abrupt reduction in the number of immature neurons between 1 (9606 ± 418 EGFP-positive cells) and 3 (4015 ± 642 EGFP-positive cells) months of age (*p* < 0.001). Analysis of additional time-points between these two ages revealed a significant decrease in the number of EGFP-positive cells between 1.5 and 2 months of age (*p* < 0.001) and between 2 and 4 months of age (*p* < 0.001). Further significant decreases in EGFP expression were detected between 4 and 12 (*p* < 0.01), 18 (*p* < 0.01), and 24 (*p* < 0.01) months of age.

In order to confirm these results, we also performed immunohistochemistry for NeuroD, a helix-loop-helix transcription factor that has been shown to be sufficient to promote neuronal differentiation of adult hippocampal neural progenitors [75] and is involved in granule cell development [76,77]. Statistical analysis revealed a significant reduction in NeuroD expression with age in the mouse hippocampal DG [one-factor ANOVA, F(9, 69) = 189.53, *p* < 0.001] (Figure 3 A-C). Again, this decrease was best fit to a negative exponential curve [*Y* = 28894x e^(-0.23X)^, with a correlation coefficient *r* ^2^ = 0.98] and a drastic reduction in the number of NeuroD-expressing immature neurons was detected between the early time-points of 1 (28334 ± 1308 NeuroD-positive cells) and 3 (12944 ± 633 NeuroD-positive cells) months of age (*p* < 0.001). When additional time-points between these two ages were analyzed, a significant reduction in the number of NeuroD-positive cells was also detected between 1.5 and 2 months of age (*p* < 0.001) and between 2 and 4 months of age (*p* < 0.001). Post-hoc analysis revealed further significant decreases in hippocampal neuronal differentiation between 4 and 12 months of age (*p* < 0.001), between 6 and 18 months of age (*p* < 0.001) and between 6 and 24 months of age (*p* < 0.001).

Of note, the fact that at any given time NeuroD expression (Figure 3A) is higher than the expression of EGFP (Figure 3D) is likely due to differences in the expression patterns of these two markers. Indeed, while EGFP expression is restricted to post-mitotic immature granule cells, peaking two weeks after mitosis and disappearing by 4 weeks [51], NeuroD is expressed in late-stage progenitor cells as they transition to the post-mitotic state and differentiate into immature granule cells (i.e., neuroblasts) [78]. Thus, while immature granule cells express both EGF and NeuroD, NeuroD is also expressed for a short period of time in late-stage progenitor cells. Nevertheless, together the EGFP and NeuroD results indicate that although neuronal differentiation occurs throughout adulthood in the mouse DG and immature neurons can still be detected at 2 years of age (48 ± 19 EGFP-positive cells and 162 ± 11 NeuroD-positive cells), there is a dramatic decrease in neuronal differentiation between 1 and 3 months of age.

### Overall hippocampal neurogenesis is significantly decreased by age

**Figure 4:**
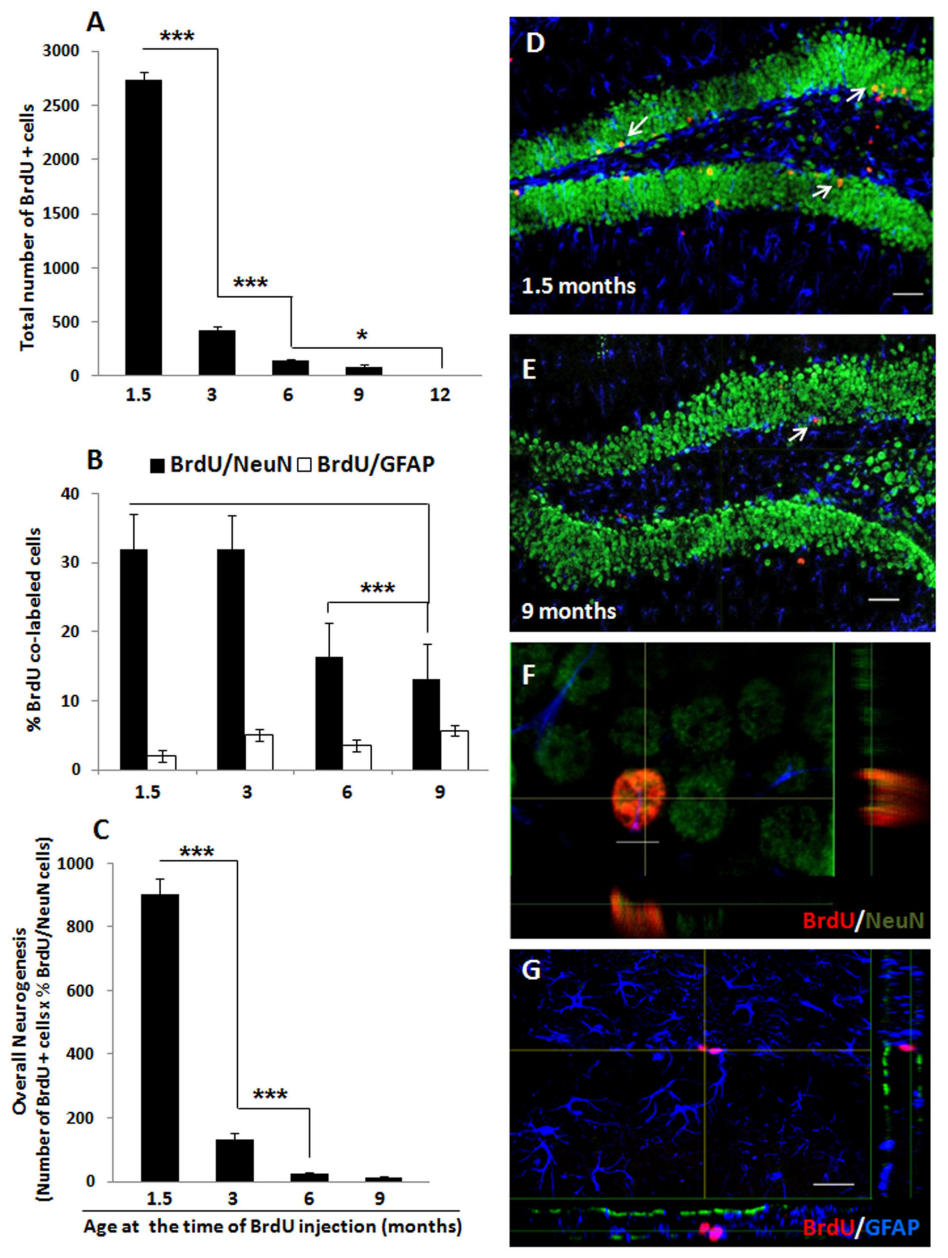
Ageing results in an abrupt decrease in overall neurogenesis in the mouse DG. C57BL/6J male mice with 1.5, 3, 6, 9, and 12 months of age received BrdU injections (200 mg/kg i.p.) every 12 hours for 3 consecutive days and were sacrificed by transcardial perfusion 42 days later, to allow enough time for differentiation of the proliferating cells that incorporated BrdU. The brains were removed, processed for BrdU/NeuN/GFAP fluorescence immunohistochemistry, and visualized by confocal microscopy. (A) A significant effect of age was found regarding the number of BrdU-labeled cells present in the DG 42 days after the last BrdU administration. * *p* < 0.05 between 6 and 12 months and between 9 and 12 months, *** *p* < 0.001 between 1.5 and 3 months and between 3 and 6 months. (B) The percentage of newly born cells (that incorporated BrdU) that co-expressed the mature neuronal marker NeuN was also significantly reduced by age while the percentage of BrdU-positive cells that co-expressed the glial marker GFAP remained unaffected by age. *** *p* < 0.001 between 1.5 and 6 or 9 months and between 3 and 6 or 9 months. (C) An estimation of overall neurogenesis based on the total number of BrdU-positive cells that survived the 42-day period multiplied by the proportion of BrdU-positive cells that acquired a mature neuronal phenotype indicates an age-induced significant and abrupt decrease in the overall number of new neurons. *** *p* < 0.001 between 1.5 and 3 months and between 3 and 6 months. (D, E) Representative confocal images of the DG showing mature granule neurons labeled with NeuN (green) and BrdU-positive cells (red) that survived over 42 days in 1.5- (D) and 9- (E) month old mice. Arrow in (E) indicates a BrdU/NeuN double-labeled cell in the DG of a 9 month-old mouse. (F, G) Orthogonal representations of immunopositive cells for (F) BrdU (red) / NeuN (green) or (G) BrdU (red) / GFAP (blue). White arrows indicate BrdU-immunopositive cells in the DG of 1.5- (D) and 9- (E) month old mice. Data are presented as means ± SEM. See text for additional statistical details. Scale bars (D, E, G) = 50 µm; scale bar (F) = 5 µm.

In order to examine how the process of ageing affects the rate of survival of newly born cells in the mouse hippocampal DG, 1.5, 3, 6, 9, and 12 month-old C57Bl/6J male mice received BrdU as outlined in Figure 1. We observed a dramatic age-related decline in the number of BrdU-labeled cells that were present 42 days after the BrdU pulse [one-factor ANOVA, F(4, 37) = 981.83, *p* < 0.001] (Figure 4A), which is consistent with previous literature [79]. Importantly, and in agreement with the proliferation (Figure 2) and differentiation (Figure 3) results, the largest decrease in survival was detected between animals that received BrdU at 1.5 months of age (2738 ± 70 BrdU-positive cells) and those that were injected with this exogenous marker at 3 months of age (423 ± 34 BrdU-positive cells; *p* < 0.001). The number of newly generated cells that survived the 42-day period continued to decrease with age with a further significant reduction noted between 3 and 6 months of age (*p* < 0.001), and no surviving cells detected in the DG of mice that received BrdU at 12 months of age (*p* < 0.01; Figure 4A).

By sacrificing the mice 42 days after the last BrdU injection, we were also able to determine the phenotypes of the new cells that incorporated BrdU. We used the mature neuronal marker NeuN and the glial marker GFAP to estimate the percentage of newly generated cells (i.e., BrdU-positive cells) that differentiated into one of these two cell types using confocal microscopy (Figure 4 B and D-G). There was an age-dependent decrease in the percentage of BrdU/NeuN double-labeled cells [one-factor ANOVA, F(3, 34) = 19.97, *p* < 0.001]. In this case, a two-fold decrease in neuronal maturation (i.e., in the percentage of BrdU/NeuN double-labeled cells) was observed between 3 and 6 months of age (mice injected with BrdU at 3 months of age: 31.9 ± 2.5% of BrdU/NeuN double-labeled cells; mice injected with BrdU at 6 months of age: 16.3 ± 2.5% of BrdU/NeuN double-labeled cells; *p* < 0.001) (Figure 4B). On the other hand, there were no significant changes in the percentage of BrdU/GFAP double-labeled cells (i.e., percentage of newly differentiated glial cells) with age [one-factor ANOVA, F(3, 34) = 1.43, *p* = 0.25] (Figure 4B).

Overall neurogenesis, calculated by multiplying the number of BrdU-positive cells that survived the 42-day period by the proportion of BrdU-positive cells that co-expressed NeuN, was also significantly reduced with age [one-factor ANOVA, F(3, 31) = 308.23, *p* < 0.001] (Figure 4C). In agreement with the proliferation (Figure 2), differentiation (Figure 3), and survival results (Figure 4A), the largest decrease in overall adult neurogenesis was observed at a very early stage (mice injected with BrdU at 1.5 months of age: 902.30 ± 48.66 new neurons; mice injected with BrdU at 3 months of age: 134.00 ± 17.73 new neurons; *p* < 0.001). A further decrease in overall adult hippocampal neurogenesis was detected between the animals that were injected with BrdU at 3 and 6 (23.82 ± 4.63 new neurons) months of age (*p* < 0.01).

### Age-associated decreases in learning

**Figure 5:**
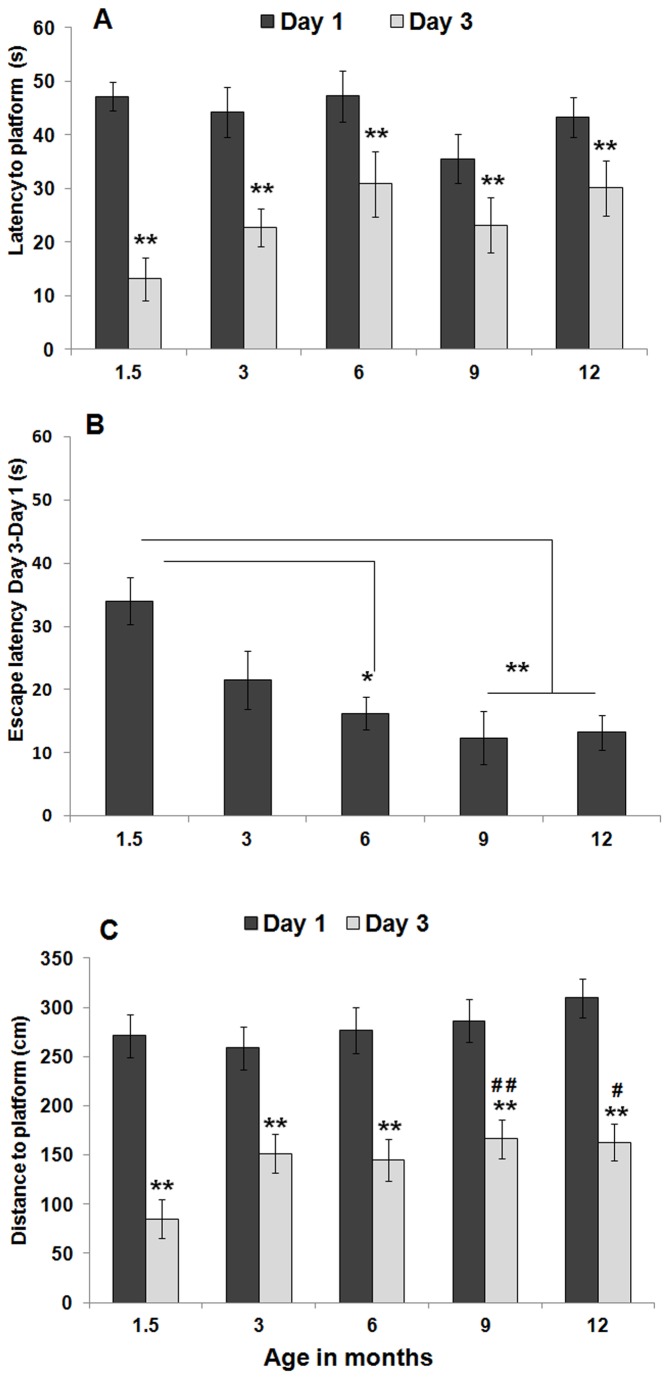
Learning of the MWM task is only modestly affected by age. C57BL/6J male mice with 1.5, 3, 6, 9, and 12 months of age were tested in the MWM for their ability to learn and recall the location of a hidden platform. (A) Time required to find the hidden platform (i.e., escape latency) in both days 1 and 3 of the MWM training. For all age groups, escape latency on day 3 of training was significantly reduced when compared with escape latency on day 1, indicating that all mice were able to learn the task. (B) When the average time required to locate the hidden platform on day 3 was subtracted from that of day 1, a significant effect of ageing on the ability to learn the MWM task was observed, with older animals taking longer to learn the location of the hidden platform. (**C**) Distance traveled to find the hidden platform (i.e., path length) in both days 1 and 3 of the MWM training. For all age groups, path length on day 3 of training was significantly reduced when compared with the respective path length on day 1, further indicating that all mice were able to learn the task. However, on day 3 of training, 1.5-month old mice swam significantly less than their oldest counterparts. Data are presented as means ± SEM. ** *p* < 0.01, when comparing day 1 and day 3 of training for each age group; ^#^ *p* < 0.05, when comparing the distance traveled on day 3 of training between 1.5- and 9-month old mice; ^##^ *p* < 0.01, when comparing the distance traveled on day 3 of training between 1.5- and 12-month old mice; see text for additional statistical details.

In order to evaluate whether normal ageing affects hippocampal-dependent spatial learning and memory, we subjected 1.5, 3, 6, 9, and 12 month-old C57Bl/6J male mice to the MWM test following a protocol similar to the one described in [19,72]. In the first part of this experiment (assessment of learning; see Figure 1), animals were allowed to learn the location of a hidden platform over a period of three consecutive days and the latency to find the platform was recorded. A factorial ANOVA revealed a significant effect of age [F(8, 68) = 3.05; *p* < 0.01]. However, further post-hoc analysis detected no significant differences in performance among the various age groups. Indeed, for each age group, a significant decrease in latency was observed when comparing performance on the first day of training (day 1) with performance on the final day of training (day 3) [repeated measures ANOVA; 1.5 months: F(1, 14) = 50.30*, p* < 0.01; 3 months: F(1, 14) = 13.48, *p* < 0.01; 6 months: F(1, 12) = 4.45, *p* < 0.05; 9 months: F(1, 14) = 3.68, *p* = 0.05; 12 months: F(1, 16) = 4.32, *p* < 0.05], indicating that all animals were able to learn the task independently of their age (Figure 5A). Nevertheless, when the average time required to locate the hidden platform on day 3 was subtracted from the average time required to locate the hidden platform on day 1 of training, a significant effect of ageing on the time required to learn the MWM task was observed [one-factor ANOVA, F(4, 35) = 5.92, *p* < 0.001]. Further post-hoc analysis revealed that 1.5 month-old mice learned significantly faster how to locate the hidden platform when compared to 6- (*p* = 0.01), 9- (*p* = 0.001), and 12- (*p* < 0.01) month old mice (Figure 5B).

Path length was also assessed for all trials of the three days of training. Again, for each age group, the distance traveled also decreased throughout the training period [repeated measures ANOVA; 1.5 months: F(1, 14) = 92.86, *p* < 0.001; 3 months: F(1, 14) = 12.82, *p* < 0.01; 6 months: F(1, 12) = 6.06, *p* < 0.05; 9 months: F(1, 14) = 29.89, *p* < 0.01; 12 months: F(1, 16) = 88.61, *p* < 0.001], further indicating that all mice acquired the task regardless of their age (Figure 5C). Similarly to latency, a significant effect of age was also observed with regards to path length [factorial ANOVA, F(8, 68) = 2.03, *p* < 0.05]. Further post-hoc analyses revealed that although all age groups traveled similar distances during the first day of training, by the last day of training the distance traveled by 1.5 month old mice was significantly shorter than that traveled by both 9- (*p* < 0.05) and 12- (*p* < 0.05) month old mice. Additionally, no significant differences between the distance traveled by 3-, 6-, 9-, and 12-month old animals were detected, indicating that from 3 months of age onwards all animals are equally effective in learning the platform location.

A factorial ANOVA also revealed a significant effect of age in the swimming speed F(8, 68) = 11.942, *p* < 0.001] with 1.5-month old mice swimming faster than their older counterparts (*p* < 0.001). No other significant differences in swimming speed were detected among 3-, 6-, 9-, and 12-month old time-points (data not shown). Since the distance traveled by 1.5 month-old mice is also reduced when compared to the distance traveled by older mice (*p* = 0.05), speed alone cannot account for the reduced latency that was observed at 1.5 months of age. Overall, these results indicate that regardless of age, all mice learn the MWM task. However, younger mice (i.e., 1.5 months old) not only swam faster, but also swam significantly less by the third day of training. Together, these results indicate that 1.5-month old mice are not only quicker but also more precise/accurate at finding the hidden platform, and therefore do not need to swim as much as their older counterparts.

**Figure 6:**
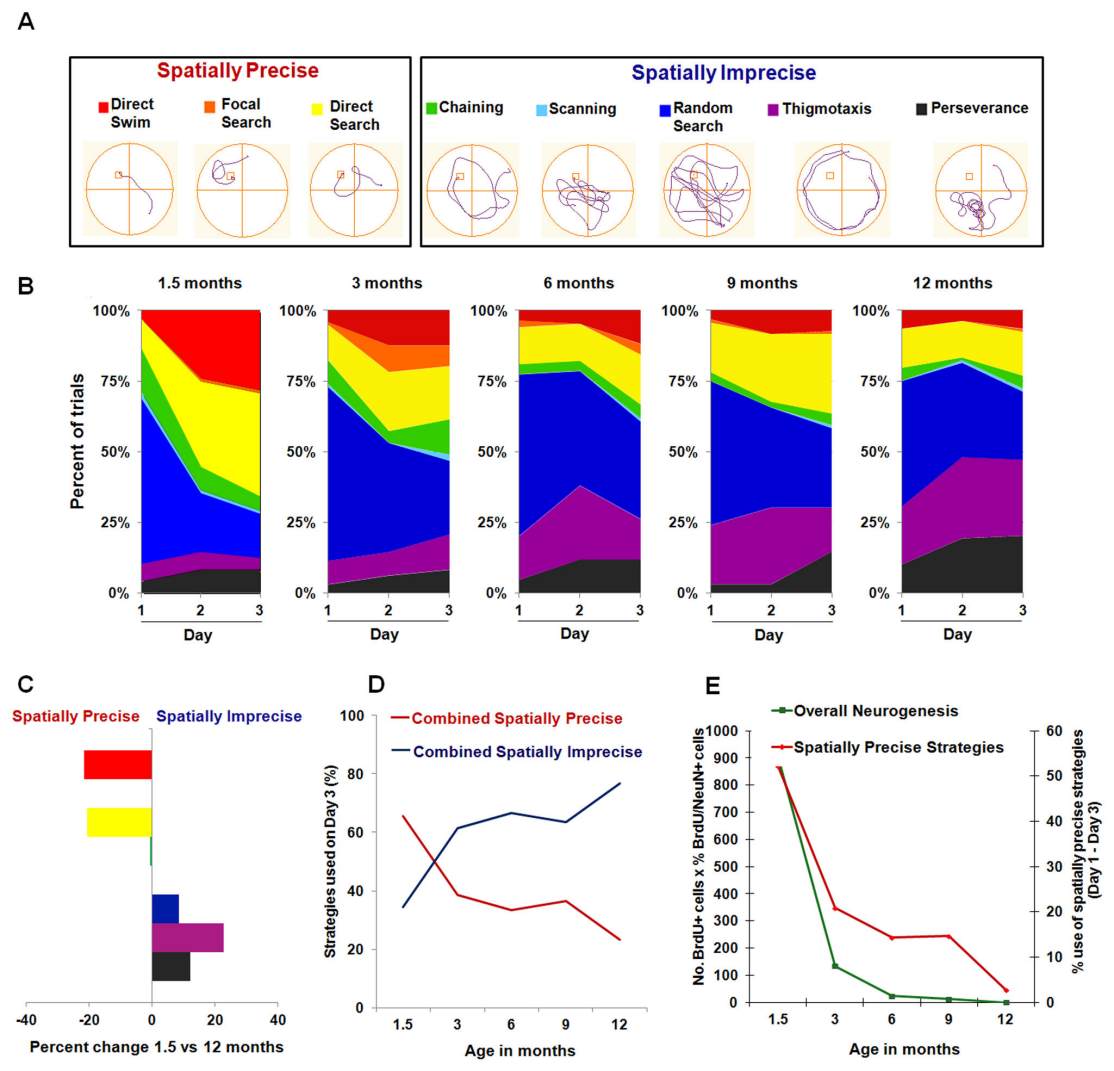
Age-induced reduction in spatially precise search strategies is correlated with levels of adult hippocampal neurogenesis. (**A**) Representative examples of the eight distinct types of search strategies employed during training in the MWM that were recognized by the classification algorithm. Strategies are grouped into either spatially precise or spatially imprecise categories. (B) Percentage of trials classified according to each search strategy across training days for the various age groups. All groups showed a progression towards an increase in the use of hippocampal-dependent spatially precise strategies with training. However, this progression clearly decreased with age. (C) Percentage difference in the frequency of each search strategy in the oldest mice (12 months old) relative to their youngest counterparts (1.5 month old). A significant reduction in the use of direct swim and direct search (spatially precise strategies) was accompanied by a significant increase in the use of random search, thigmotaxis, and perseverance (spatially imprecise strategies) in 12 month-old mice. (D) Age resulted in a decrease in the combined use of spatially precise search strategies and a concomitant increase in the combined use of spatially imprecise search strategies. (E) The use of spatially precise search strategies (calculated as the % use of combined spatially precise strategies on day 3 of training – day 1 of training) is strongly correlated with the levels of overall hippocampal neurogenesis (calculated as in Figure 4C). Correlation coefficient *r* ^2^ = 0.965. See text for additional statistical details.

Indeed, both spatially precise and imprecise search strategies can contribute to a reduction in escape latency over the three days of the training period [80-83]. Thus, we also performed a detailed analysis of the search strategies employed during the three days of training using tracking data and a criterion-based algorithm [64,74]. Individual search strategies were objectively classified into eight mutually exclusive categories (Section 2.5; Figure 6A). At the beginning of training (day 1), spatial-independent strategies (mainly random search, perseverance, and thigmotaxis) predominated in all age groups, accounting for approximately 80% of the adopted paths (Figure 6B). However, for each age group, these spatially imprecise strategies were progressively replaced by more localized/spatially precise strategies throughout the three-day training period [Friedman’s ANOVA; 1.5 months: 2(8) = 20.68, *p* < 0.01; 3 months: 2(8) = 21.60, *p* < 0.01; 6 months: 2(8) = 23.66, *p* < 0.01; 9 months: 2(8) = 23.37, *p* < 0.01; 12 months: 2(8) = 23.51, *p* < 0.01]. Spearman Rank Order Correlations revealed that for all age groups a specific decrease in the frequencies of perseverance (*p* < 0.05) and random search (*p* < 0.001) was accompanied by a specific increase in the frequencies of direct search (*p* < 0.05) and direct swimming (*p* < 0.01) with training. Spearman Rank Order Correlations also revealed a specific increase in the frequency of thigmotaxis (*p* < 0.001) with age (Figure 6B). In particular, when comparing the search strategies used in the last day of training by the youngest and oldest mice (i.e., 1.5 and 12 month-old animals), it becomes clear that while young mice preferentially learn through the use of spatially precise search strategies (namely direct swim and direct search), the oldest animals continue to rely on spatially imprecise strategies (random search, thigmotaxis, and perseverance) on the last day of training (Figure 6C).

We have also grouped the various search strategies into either spatially precise or imprecise categories (Figure 6A) and determined the percentage of use of both categories at the end of the training period (i.e., day 3) (Figure 6D). Strikingly, we observed a clear decrease in the cumulative use of spatially precise strategies and a concomitant increase in the cumulative use of spatially imprecise strategies with age (Figure 6D). These analyses indicate that in young mice, learning is preferentially mediated by the adoption of localized/spatially precise search strategies and that the ability to use this type of strategies clearly decreases with age. Indeed, while the cumulative use of spatially precise search strategies increases by 52.1% between days 1 and 3 of training for 1.5 month-old mice, for the remaining age groups this increase is not as accentuated (3 months: 20.8%; 6 months: 14.3%; 9 months: 14.6%; 12 months: 2.8%). Importantly, this reduction in the ability to adopt spatially precise strategies with age is strongly correlated with the age-related decrease in overall hippocampal neurogenesis depicted in Figure 4C (correlation coefficient *r* ^2^ = 0.97; Figure 6E). Additionally, a strong correlation between the levels of overall neurogenesis and the % use of spatially precise strategies on the last day of training (day 3) was also found (correlation coefficient *r* ^2^ = 0.93) (data not shown). Furthermore, a Pearson Correlation Analysis that takes into account the levels of overall neurogenesis and the use of spatially precise strategies by each individual animal revealed that the Pearson Correlation Coefficient between these two parameters was *r* = 0.81. This analysis also revealed significant Pearson Correlation Coefficients between age and the individual levels of overall neurogenesis (*r* = -0.65) as well as between age and the individual use of spatially precise strategies (*r* = -0.63). These additional statistical analyses further indicate that with age the levels of overall neurogenesis are highly correlated with the use of spatially precise search strategies.

### Age-associated decreases in memory

Memory (i.e., the ability to remember the location of the platform) was evaluated through a 60 second probe-test performed 28 days after assessment of learning (see Figure 1 and Section 2.5 for experimental details). Of note, during the probe trial mice were placed on the platform for 15 seconds before being tested in the absence of the platform as previously described [19,72] (see Section 2.5 for experimental details). Thus, to rule out the possibility of “new learning” contributing to performance during the probe test, the distance traveled to reach the platform on trial 1 of day 1 of training (i.e., when mice were being tested in the MWM for the first time and were also placed on the platform for 15 seconds at the beginning of the trial) was compared with the distance traveled to reach the exact area where the platform used to be located during the probe trial. For all age groups, the distance traveled during the first trial of the first day of training was significantly longer than the distance traveled during the probe trial (Student’s t test; *p* < 0.001 for all age groups). Thus, a single 15 seconds exposure to the platform was not enough for animals to “memorize” its location (as in the case of the first trial of the first day of training), and repeated exposure to the platform over three days of training (12 trials/day) was required for memory formation. Thus, we believe this analysis further validates the use of this probe trial (originally described by 19) as a test for memory rather than learning. Nevertheless, the brief 15 seconds exposure to the platform immediately before the probe test is likely to have facilitated memory recall and thus influenced the precision with which spatial navigation occurred during the probe test. However, since all animals were submitted to the same behavioral paradigm, it is unlikely that this aspect of the test would have significantly influenced group differences.

**Figure 7:**
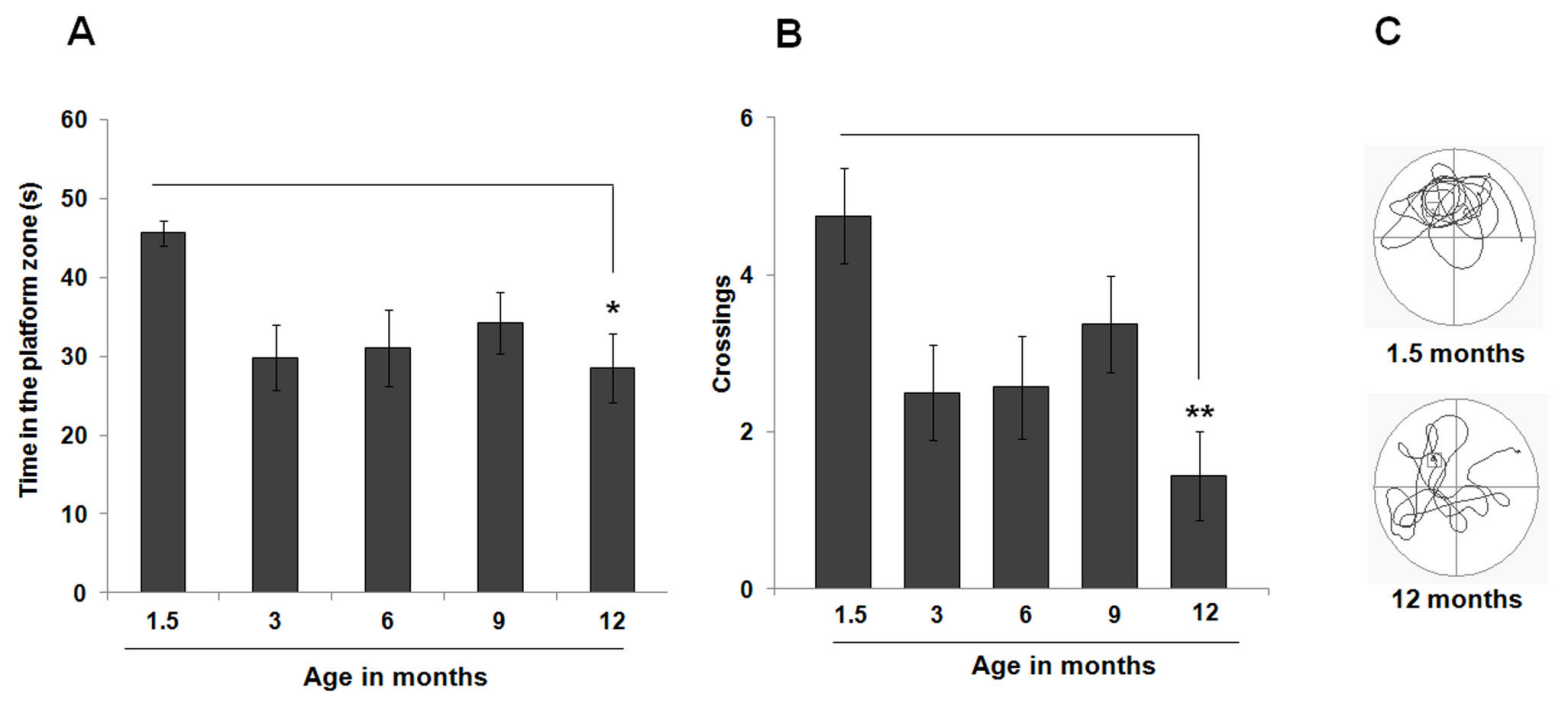
Memory is only modestly affected by age. A significant effect of age on the performance of the probe test was observed, with 1.5-month old mice spending significantly more time in the target quadrant (A) and crossing the platform zone more often (B) than their oldest counterparts. (C) Representative examples of swim paths of 1.5- and 12-month old mice during the probe test. Although both mice swam in the quadrant where the platform used to be located, the younger animal swam predominately more in the target quadrant. Data are presented as means ± SEM. * *p* < 0.05, ** *p* < 0.01, see text for additional statistical details.

The total time spent in the NW quadrant of the pool (where the platform used to be located) during the 60-second test was measured and evidence of memory was found whenever an animal spent more than 25% of time (i.e., at least 15 seconds) in the target quadrant. All age groups showed evidence of memory as assessed with a one-sample t-test applied against this “chance” value (1.5 months: 45.63 ± 1.62 seconds, *p* < 0.001; 3 months: 29.79 ± 4.13 seconds, *p* < 0.01; 6 months: 31.02 ± 4.88 seconds, *p* < 0.05; 9 months: 34.25 ± 3.83 seconds, *p* < 0.01; 12 months: 28.57 ± 4.38 seconds, *p* < 0.05). Nevertheless, a significant effect of age was observed [one-factor ANOVA, F(4, 35) = 3.14, *p* < 0.05], with 1.5-month old mice spending significantly more time in the target quadrant than their 3- (*p* = 0.05), and 12- (*p* < 0.05) month old counterparts (Figure 7A). Additionally, a significant correlation between the levels of overall neurogenesis and the performance in the probe test was also obtained (*r* ^2^ = 0.93). Furthermore, a Pearson Correlation Analysis that takes into account the levels of overall neurogenesis and the performance during the probe test by each individual animal revealed that the Pearson Correlation Coefficient between these two parameters was *r* = 0.50. Additionally, this analysis also revealed a significant Pearson correlation coefficient between age and the individual performances in the probe test (*r* = -0.28). These additional statistical analyses suggest that with age, the levels of neurogenesis may also influence memory performance, albeit not to the same degree as performance during the learning phase of the MWM. Nevertheless, these significant correlations further highlight that the peak in performance during the probe test occurs when overall neurogenesis is at its highest (i.e., at 1.5 months of age).

A significant effect of age on the number of crossings (i.e., number of times each animal crossed the platform zone) was also detected [one-factor ANOVA, F(4, 35) = 4.19, *p* < 0.01], with a significant increase observed in 1.5-month old mice (4.75 ± 0.61 crossings) as compared with their 12-month old counterparts (1.44 ± 0.57 crossings; *p* < 0.01) (Figure 7B).

## Discussion

In the present study we show that there is a progressive age-related decline in all stages of the neurogenic process in the mouse hippocampus. In agreement with previous studies [48,84], we found that this age-induced reduction in adult hippocampal neurogenesis does not occur at a constant rate throughout the mouse life span, fitting instead an exponential decay model. Interestingly, while the initial sharp and abrupt decrease in adult hippocampal neurogenesis that occurs between 1.5 and 3 months of age is paralleled by a significant reduction in MWM learning (i.e., escape latency and path length) and memory (i.e., time spent in the target quadrant, and number of crossings through the platform zone), further reductions in neurogenesis are not accompanied by equivalent decreases in these parameters. However, a detailed analysis of the adopted search strategies revealed a strong correlation between the use of spatially precise strategies and overall neurogenesis with age (Figure 6E).

### Aging induces an abrupt decrease in adult hippocampal neurogenesis

It is clear that age negatively correlates with endogenous neurogenic activity in mammals, including mice [48,79,85-92], rats [6,26,50,93,94], lesser hedgehog tenrecs [95], marmosets [96], tree shrews [97], red foxes [98], dogs [99], macaques [100] and humans [101]. A detailed analysis by Lazic [84] revealed that for most cases this age-related reduction in hippocampal neurogenesis is best fit to a negative exponential model with relatively few new cells being added during an animal’s life, and only a proportion of these maturing into functional neurons. Moreover, the rate of decrease in neurogenesis appears to correlate with species longevity but not with their body mass or basal metabolic rate [84].

In this study we extended these findings and systematically characterized changes in the various phases of the neurogenic process across the entire mouse life span (i.e., up to 24 months of age). In agreement with recent studies [48,84], the age-related decrease in hippocampal neurogenesis did not follow a linear pattern, fitting instead a negative exponential model characterized by an initial abrupt reduction in cell proliferation and neuronal differentiation between 1.5 and 3 months of age. Specifically, there was an 80% and 53% reduction in the number of cells expressing the endogenous proliferation markers Ki-67 and PCNA respectively (Figure 2). Similarly, between 1.5 and 3 months of age the expression of the neuronal differentiation markers NeuroD and EGFP was decreased by 56% and 58% respectively (Figure 3). By the time animals reached 24 months of age, hippocampal neurogenic capacity was virtually abolished, with a 98-99% reduction in the expression of all proliferation (i.e., PCNA and Ki-67; Figure 2) and differentiation (i.e., NeuroD and EGFP; Figure 3) markers. Although no other study has systematically analyzed the mouse hippocampal neurogenic capacity up to 24 months of age, cell proliferation has been reported to decrease by 85% between the ages of 3 and 9 months [48] and by 90% between the ages of 2 and 12 months [85] in C57Bl/6J mice. Moreover, significant age-induced decreases in the expression of doublecortin (DCX), a commonly used marker of immature neuroblasts [102], have also been observed [48,88], further validating the results presented here.

We also determined the percentage of new cells that became mature neurons at 1.5, 3, 6, 9 and 12 months of age. We found a 48% decrease in the percentage of BrdU/NeuN double-labeled cells between 3 and 6 months in the hippocampus of C57BL/6J mice (Figure 4B), indicative of an age-related decrease in the rate of neuronal differentiation. In contrast to these results, Ben Abdallah and co-workers [48] calculated the ratio between the number of Ki-67- and DCX-positive cells as an estimation of neuronal differentiation rate and found no changes between 1 and 9 months of age [48]. Differences in the expression pattern of the markers used are likely to account for these discrepancies. Indeed, while Ki-67 is an endogenous marker that is expressed during all active phases of the cell cycle but is absent from cells at rest [54,55], BrdU is only incorporated into the DNA double-helix of replicating cells during the S-phase of the cell cycle, remaining in those cells once they become post-mitotic [58]. On the other hand, while DCX is expressed only transiently in neuroblasts migrating from the SGZ into the GCL and its expression falls below detectable levels before neurons become mature [102], NeuN is expressed in terminally differentiated post-migratory neurons [10]. Thus, while the ratio between the number of Ki-67- and DCX-positive cells gives an indication of the proportion of dividing cells that assumed neuronal phenotypes [48], the ratio of BrdU/NeuN double-labeled cells reflects the proportion of newly generated cells that actually became mature neurons. Furthermore, a percentage of newly generated cells is reported to die within a 4 week period [25,103] and therefore, the proportion of BrdU/NeuN double-labeled cells calculated 42 days after the BrdU pulse provides a more accurate estimation of the proportion of newly generated cells that survived to become fully differentiated neurons.

We found the percentage of newborn cells expressing the astroglial marker GFAP to be unchanged with age (Figure 4B). This is in contrast to one study that reported an increase in gliogenesis between 6- and 18-month old rats [104], however other studies have also found gliogenesis to be unchanged across the lifespan both in rats [105] and mice [79]. Our study used a younger age range with a finer temporal resolution. Thus, although gliogenesis rates may change with age, our results show this process occurs at relatively constant levels until at least 12 months of age in the mouse DG. It should be noted however that GFAP is also expressed by type-1 putative stem cells [10,67,106]. Therefore we cannot exclude the possibility that some portion of GFAP-positive cells counted were in fact radial glia-like cells which may in turn give rise to pluripotent hippocampal precursor cells [10,67,106].

When estimating overall hippocampal neurogenesis, we found a massive decline of 85% in neurogenic capacity between 1.5 and 3 months of age (Figure 4C). This is likely a result of the massive decrease in cell proliferation (Figure 2) and neuronal differentiation (Figure 3) that occur during this period in the mouse DG. However, while cell proliferation (Figure 2) and neuronal differentiation (Figure 3) were detectable up to 24 months of age, overall neurogenesis was found to be virtually abolished by 9-12 months (Figure 4 C), as a consequence of the drastic reduction in the survival of new cells that is observed around this time (Figure 4A). Of note, while direct comparisons between the results on cell proliferation (Figure 2) and neuronal differentiation (Figure 3) obtained in naïve mice and the results on cell survival/neuronal maturation/overall neurogenesis (Figure 4) obtained in trained mice should not be made, it is clear that age affects the neurogenic process in a similar way, both in naïve (Figures 2 and 3) and trained (Figure 4) mice. As a matter of fact, while cell proliferation (Figure 2) and neuronal differentiation (Figure 4), which were determined in naïve mice, can still be detected up to 24 months of age, cell survival (Figure 4A) and overall neurogenesis (Figure 4C), which were determined in trained mice, can only be detected up to 9 months of age. Thus, it is possible to infer that the effects of MWM training on neurogenesis in the second cohort of mice were not robust, as their neurogenic capacity did not extend beyond 9 months of age. Furthermore, the abrupt decrease in cell proliferation (Figure 2) and neuronal differentiation (Figure 3) that was noted between 1.5 and 3 months of age in naïve mice was also mirrored by a similar abrupt reduction in cell survival (Figure 4A) and overall neurogenesis (Figure 4C) in trained mice. Thus, even though we cannot exclude the possibility that MWM training might have enhanced neurogenesis during the earlier time points, the effect of training did not alter the normal time-course of the effect of age on neurogenesis.

### The age-related decrease in overall hippocampal neurogenesis parallels a reduction in the use of spatially precise search strategies

A number of studies have shown that hippocampal neurogenesis is positively correlated with increased learning and behavioural experience [25-27]. Conversely, it has also been shown that reductions in neurogenic function can impair memory formation [20,28-31]. However, to date no study has systematically investigated how these two hippocampal-related phenomena vary with age. As such, in this study we used the same cohort of animals to assess the effects of ageing not only on overall hippocampal neurogenesis but also on hippocampal-dependent learning and memory (Figure 1).

We found that all age groups showed evidence of learning (Figure 5A and C) and memory (Figure 7A) as assessed by the MWM test. Nevertheless, during the training period (i.e., acquisition phase), a significant reduction in escape latency (Figure 5B) and distance traveled (Figure 5C) was detected in 1.5-month old mice when compared to older mice. Moreover, the youngest animals spent considerably more time in the target quadrant (Figure 7A) and crossed more times the platform zone (Figure 7B) during the probe test when compared to their older counterparts. However, although this peak in performance occurs concomitantly with the peak in hippocampal cell proliferation (Figure 2), neuronal differentiation (Figure 3), and overall neurogenesis (Figure 4C), the exponential decrease in neurogenesis that was observed with age was not mirrored by a similar decrease in these parameters of the MWM task. These findings are in agreement with a recent study by Martinez-Canabal and colleagues (2013) who showed that the effects of inhibiting hippocampal neurogenesis on MWM performance (i.e., escape latency) are age dependent. These authors showed that while suppressing neurogenesis impaired the performance of juvenile mice (1-2 months old), it did not have an effect on the performance of adult mice (2-3 months old) and middle-aged mice (11-12 months old) [107].

Indeed, previous studies have shown that while escape latency may not be affected by adult neurogenesis, the use of spatially precise strategies is an aspect of MWM learning that is highly dependent on the neurogenic capacity [64,74]. As such, in this study we also performed a detailed analysis of the search strategies employed during the three days of MWM training by the various age groups (Figure 6). We found that while the use of more spatially precise strategies increased for all age groups over the training period (Figure 6B), there was a clear decrease in the use of these more localized strategies with age (Figure 6D). Furthermore, this age-related reduction in the ability to employ spatially precise search strategies is highly correlated with the levels of overall hippocampal neurogenesis (Figure 6E), strongly suggesting that these two events are indeed related. In fact, while at 1.5 months of age the peak in overall hippocampal neurogenesis is accompanied by a similar peak in the use of spatially precise search strategies, at 12 months of age, when overall hippocampal neurogenesis is virtually abolished, the use of spatially precise strategies during the training period is minimal (Figure 6 C-E).

These findings also show that as mice age, the use of more spatially imprecise search strategies become more predominant during learning (Figure 6D). Since no significant differences in escape latency (Figure 5 A-B) and path length (Figure 5C) were observed with age beyond 1.5 months of age, these results suggest that while the levels of overall hippocampal neurogenesis may not impact learning ability (i.e., escape latency and path length) beyond the initial peak in performance (Figure 5), they may dictate the way animals learn this task by favoring the use of spatially precise versus spatially imprecise search strategies (Figure 6 D-E).

On the other hand, the fact that at 1.5 months of age, when overall neurogenesis peaks (Figure 4C), mice show the greatest reduction in escape latency and path length with training (Figure 5B-C), suggests that early on in life the elevated rate of neurogenesis might influence not only the way animals learn the task (i.e., through the use of more localized search strategies), but also their ability to learn the task faster. However, we cannot rule out the possibility that the increase in swimming speed (i.e., an indicator of better locomotor performance) detected at 1.5 months of age underlies the reduced escape latency observed at this young age. Nevertheless, the fact that 1.5 month-old mice also show a reduction in path length suggests that speed alone cannot explain the decrease in latency and that these young mice not only swam faster, but also swam less (i.e., more efficiently) than their older counterparts. Furthermore, this decrease in the distance traveled detected at 1.5 months of age is in line with the use of more spatially precise (i.e., efficient) search strategies that require less swimming (i.e., shorter path lengths) by the youngest mice (Figure 6B).

Interestingly, while the type of search strategies employed continues being affected by the levels of hippocampal neurogenesis with age (Figure 6E), the ability to learn the task (i.e., escape latency) is not further influenced by the age-induced decrease in neurogenesis after 1.5 months of age (Figure 5). Thus, the peak in hippocampal neurogenesis that occurs at around 1.5 months of age may result in faster learning (i.e., reduced escape latency and distance traveled with increased use of spatially precise search strategies) and better memory performance (i.e., more time spent in the target quadrant during the probe test). However, with age pre-existing neurons may compensate for the reduction in neurogenesis in such a way that the ability to learn the MWM task later on in life is relatively preserved despite the age-induced decrease in the use of neurogenesis-dependent spatially precise search strategies.

It is also possible that the initial peak in adult hippocampal neurogenesis that occurs early on in life may contribute to a neural reserve and the maintenance of functional plasticity and behavioral performance, thus compensating for situations of functional loss that occur with ageing [108]. These hypotheses are in agreement with previous studies showing that the effect of MWM training on cell proliferation and survival is not a simple case of “more is better”. Indeed, Epp and colleagues [109] found decreased DG cell survival with increasing MWM task difficulty, despite animals still being able to perform the tasks adequately [109]. In a different study, the authors found that inhibiting neurogenesis using a telomerase inhibitor could actually improve performance in the MWM [110]. Together, these studies indicate that the relationship between the creation of new neurons and learning and memory ability is far from linear. Nevertheless, our results strongly suggest that certain aspects of spatial learning, such as the use of spatially precise search strategies, are dependent on the levels of hippocampal neurogenesis (Figure 6E).

Of note, a few studies have also analyzed how hippocampal neurogenesis and learning and memory vary with age in rats [111,112]. Bizon and colleagues [111] have compared neurogenesis levels and MWM performance in 7- and 25-month old rats and found that in the older animals higher numbers of BrdU-positive cells were associated with worse performance in the learning task (as assessed by the number of search errors and spatial learning index). However, in that study BrdU was administered after animals were submitted to behavioural testing [111], and not before (as in the present study; see Figure 1), and thus it is not possible to conclude on the effects of MWM learning on neurogenesis during the actual exposure to behavioural testing. Indeed, in aged rats the influence of spatial learning on the survival of newly born cells was shown to be highly dependent on the birthdate of these new cells. Thus, while learning increases the survival of cells generated before learning, it decreases survival of cells produced during the early phase of learning [112]. On the other hand, it is important to note that there are substantial differences in hippocampal structural and functional plasticity between rats and mice [113]. In adult rats newly born neurons express mature neuronal markers and activity-induced immediate early genes 1-2 weeks earlier than those in mice. Furthermore, the number of newly born cells that survive and functionally integrate into hippocampal learning circuits also appears to be higher in rats than in mice [114], and therefore direct comparisons between these two species should be avoided. Nevertheless, overall these studies indicate that aged rats are also able to maintain cognitive function despite pronounced reductions in hippocampal neurogenesis [111,112]. Additionally, recent studies have indicated that both daily exercise [114] and environmental enrichment [115] can improve hippocampal-dependent spatial learning and memory in aged rats by modulating hippocampal neurogenesis [114,115] and neuroimmune cytokine signaling [114]. Although these studies did not perform a detailed analysis of the search strategies used by the aged rats in the MWM, it may be that physical exercise and/or environmental enrichment favor the use of more spatially precise search strategies (and a concomitant decrease in the use of more spatially imprecise strategies). This possibility in turn corroborates the beneficial effects that have been repeatedly reported in the clinical setting with both physical exercise and environmental enrichment and further supports the use of these non-invasive strategies for the treatment of age-induced cognitive deficits.

Finally, it is important to note that assessment of spatial learning and memory through the MWM test alone might not be the most sensitive method to evaluate whether the age-related decrease in hippocampal neurogenesis affects cognition [116]. Recently, Sahay and colleagues [117] have used genetic manipulations to selectively increase the survival of newborn neurons in the hippocampus and found that more neurogenesis resulted in an increase in the ability to discriminate between two similar contexts, suggesting improved pattern separation. However, the authors observed no differences between animals with normal and increased levels of hippocampal neurogenesis with regard to their performance in other behavioral tests that have been correlated with neurogenic activity including the MWM [117]. Nevertheless, these authors did not conduct a detailed analysis of the types of search strategies used by the animals in the MWM. Thus, it is unclear whether increased neurogenesis would have resulted in a shift towards the use of more spatially precise strategies, which would be expected based on the specialized role of the DG in generating a metric rather than just a configurational map of the environment [64]. Nevertheless, future studies analyzing the effects of normal ageing on context discrimination and pattern separation are warranted.

## Conclusion

Ageing is one of several factors that negatively modulate the neurogenic capacity of the adult mammalian brain. However, this age-induced decline in adult hippocampal neurogenesis does not occur at a constant rate. Instead, a sharp and abrupt reduction in overall neurogenesis occurs early on in adulthood. This phenomenon is mirrored by a significant decrease in the use of spatially precise search strategies during MWM learning. However, although hippocampal neurogenesis and overall learning and memory abilities peak simultaneously early on in life, these aspects of cognition remain relatively unaffected by age, despite the decrease in neurogenesis and the reduced use of spatially precise search strategies, since animals can adopt other strategies to compensate for these deficits. Together, these results suggest that alternate spatially imprecise search strategies may allow the aged brain to retain a moderate capacity for learning spatial tasks.
